# Impact of MLC properties and IMRT technique in meningioma and head-and-neck treatments

**DOI:** 10.1186/s13014-015-0447-z

**Published:** 2015-09-02

**Authors:** Steffi Kantz, Matthias Söhn, Almut Troeller, Michael Reiner, Helmut Weingandt, Markus Alber, Claus Belka, Ute Ganswindt

**Affiliations:** Department of Radiation Oncology, Ludwig-Maximilians-University, Munich, Germany; Department of Radiation Oncology, William Beaumont Health System, Royal Oak, MI USA; Department of Clinical Medicine, Department of Oncology, Aarhus University, Aarhus, Denmark

**Keywords:** MLC properties, IMRT, VMAT

## Abstract

**Purpose:**

The impact of multileaf collimator (MLC) design and IMRT technique on plan quality and delivery improvements for head-and-neck and meningioma patients is compared in a planning study.

**Material and methods:**

Ten previously treated patients (5 head-and-neck, 5 meningioma) were re-planned for step-and-shoot IMRT (ssIMRT), sliding window IMRT (dMLC) and VMAT using the MLCi2 without (−) and with (+) interdigitation and the Agility-MLC attached to an Elekta 6MV linac. This results in nine plans per patient. Consistent patient individual optimization parameters are used. Plans are generated using the research tool Hyperion V2.4 (equivalent to Elekta Monaco 3.2) with hard constraints for critical structures and objectives for target structures. For VMAT plans, the improved segment shape optimization is used.

Critical structures are evaluated based on QUANTEC criteria. PTV coverage is compared by EUD, D_mean_, homogeneity and conformity. Additionally, MU/plan, treatment times and number of segments are evaluated.

**Results:**

As constrained optimization is used, all plans fulfill the hard constraints. Doses to critical structures do not differ more than 1Gy between the nine generated plans for each patient. Only larynx, parotids and eyes differ up to 1.5Gy (D_mean_ or D_max_) or 7 % (volume-constraint) due to (1) increased scatter, (2) not avoiding structures when using the full range of gantry rotation and (3) improved leaf sequencing with advanced segment shape optimization for VMAT plans. EUD, D_mean_, homogeneity and conformity are improved using the Agility-MLC. However, PTV coverage is more affected by technique. MU increase with the use of dMLC and VMAT, while the MU are reduced by using the Agility-MLC. Fastest treatments are always achieved using Agility-MLC, especially in combination with VMAT.

**Conclusion:**

Fastest treatments with the best PTV coverage are found for VMAT plans with Agility-MLC, achieving the same sparing of healthy tissue compared to the other combinations of ssIMRT, dMLC and VMAT with either MLCi2^−/+^ or Agility.

## Introduction

The major vendors of clinically used linear accelerators (linacs) re-designed their multi-leaf-collimators (MLCs) over time as importance of the leaf width, transmission, maximum leaf speed and leaf positioning accuracy as well as interdigitation capabilities was investigated in the literature.

Burmeister *et al.* [1] and Wu *et al.* [2] demonstrated that leaf width has only small impact on the plan quality for large PTVs. Benefits of smaller leaf width and leaf penumbra are predominantly encountered in cases of small lesions and complex PTV or organs at risks (OAR) [3–6]. Furthermore, smaller leaves may reduce radiation to surrounding tissue by up to 5 % for the 70 and 50 % isodose [4]. Bortfeld *et al.* [7] showed that the optimal leaf width is 1.5–1.8 mm when neglecting transmission.

On the other hand, Topolniak *et al.* [5] showed transmission to be one of the most important parameters towards higher quality plans. As lower transmission means lower dose to surrounding tissues, either less dose to healthy tissue or higher modulation degrees become feasible.

The importance of leaf positioning accuracy was demonstrated for various MLC designs, showing that leaf positioning errors need to be considered particularly when the leaves are moving at maximum speed and/or for highly modulated MLC patterns [8–11]. Furthermore, Low [8] and Vorwerk [12] proved the dependency of treatment time on leaf speed limitation. Thereby, not only the positioning accuracy, but also the velocity of the leaves is important for high quality plan delivery.

Concerning MLC interdigitation, literature reveals contradictory findings. Webb [13] describes this feature as favorable and Tacke *et al.* [14] show faster delivery times for complex step-and-shoot IMRT treatment plans (head-and neck, prostate including lymph nodes). For VMAT treatments, Van Kesteren *et al.* [15] show no improvement for prostate and rectum treatment plans whereas Lafond *et al.* [16] report at least improvements in delivery efficiency.

While Varian (Varian Medical Systems, Palo Alto, CA, USA) was the first company to introduce a MLC with a leaf width of 5 mm for large fields (40 × 40 cm^2^)– at least for the inner 20 cm –, Siemens (Siemens Medical Solutions, Germany) was then the first to provide a MLC with 5 mm leaf width over the whole 40 × 40 cm^2^ field [14]. Besides the Beam Modulator™ MLC (4 mm leaves over a maximal field size of 21 × 16 cm^2^) and the MLCi2 (10 mm leaves over the whole field size of 40 × 40 cm^2^), Elekta (Elekta AB, Stockholm, Sweden) also introduced a MLC with 5 mm leaves over a 40 × 40 cm^2^ field size (Agility™-MLC). Compared to the Beam-Modulator-MLC and the MLCi2, the Agility-MLC has lower transmission, as well as improved tongue-and-grove-effect and penumbra [17].

Numerous studies exist that evaluate the impact of some specific MLC design parameters on specific delivery techniques or the impact of different delivery techniques on certain treatment sites based on miscellaneous physical and clinical parameters of the dose distribution. As the various studies used diverse treatment planning approaches, optimization and dose calculation algorithms, the relationship of MLC design, delivery technique, optimization approach and treatment site is hard to obtain. To facilitate conclusions about the influence of different MLC design parameters with regard to different IMRT techniques for complex treatment volumes, this work presents a consistent planning study comparing three different MLC designs used with three different IMRT techniques for two treatment sites, by using a single treatment planning system.

The influence on clinical relevant parameters (target coverage, dose to critical structures, monitor units per plan and treatment times) is shown for different MLC designs by means of the Elekta MLCi2-MLC (without (−) and with (+) interdigitation) and the Elekta Agility-MLC and for different IMRT techniques by means of step-and-shoot IMRT (ssIMRT), dynamic-sliding window IMRT (dMLC) and volumetric modulated arc therapy (VMAT). For this comparison, plans were generated for meningioma and head-and-neck cases, using the treatment planning software Hyperion V2.4 (University of Tübingen, Germany, research version of Elekta MONACO 3.2) that employs constrained optimization and a Monte Carlo dose algorithm.

## Material and methods

A planning study was performed for five head-and-neck (HN) and five meningioma (MG) cases. For each case, nine plans were generated: three step-and-shoot IMRT (ssIMRT), three dynamic-sliding-window-IMRT (dMLC) and three volumetric modulated arc therapy (VMAT) plans, with each technique using the MLCi2-MLC with and without interdigitation (MLCi2^+^, MLCi2^−^) and the Agility-MLC. All plans were generated for an Elekta 6MV linac.

### Treatment planning system

Hyperion V2.4 (University Tübingen, Germany, research version of Elekta MONACO 3.2) was used as treatment planning system [18–20]. It uses constrained optimization, *i.e.* at all stages of optimization all dose-limiting constraints are ensured to be strictly fulfilled. Hence, in constrained optimization, PTV coverage is an objective that will be fulfilled only, if the dose-limiting constraints allow for that. The functions applied in the optimization may not only be physical constraints (*e.g.* dose-volume-constraints, quadratic overdose, *etc.*) but also functions modelling the biological effect of radiation to different tissues (*e.g.* serial or parallel equivalent uniform dose, EUD) as described by Alber *et al.* [21, 22] The optimization is implemented as a two-step approach: First, the fluence matrix is optimized using an advanced pencil beam algorithm [23, 24]. Second, the fluence matrix is segmented into an initial-guess MLC-sequence and subsequently optimized employing a segment shape and weight optimization [25]. Final dose calculation is performed with the XVMC Monte-Carlo dose engine [26].

VMAT optimization includes the use of the recently introduced advanced segment shape optimization, as part of the second stage optimization algorithm. During the first optimization stage, VMAT fluences are obtained on equidistant, user-defined gantry angles (15 during this study). Sequencing translates the fluence maps to deliverable segments at all available angles as sets of control points with definition of gantry angle, dose as MU for the segment and position for every leaf and jaw. Due to the rotation around the patient, differences in the OAR-to-PTV projection occur between the 15 equidistant gantry sampling points used during the fluence optimization and the continuous gantry positions used after the sequencing step. These are accounted for by the advanced segment shape optimization by checking every MLC position for each segment with regard to its influence on the OAR constraints and the PTV objectives. Thereby each leaf is opened or closed iteratively to find its optimal position.

### MLC properties

The main properties of a MLC are the leaf width, maximum speed and minimal gap between opposing leaves as well as interdigitation capabilities. Table 1 shows all relevant differences between the MLCi2-MLC and the Agility-MLC. Generally, the smaller the leaf width, the better the field shape can be conformed with respect to the PTV and OARs. This potentially results in better OAR-sparing while the PTV coverage is maintained. A minimal leaf gap between two opposing leaves needs to be maintained for dynamic techniques to prevent colliding leaves. On the other hand, radiation passes through this gap and causes unwanted dose to tissue and reduces thereby some degree of freedom in the optimization process. Therefore, a small leaf gap with low radiation passing through is wanted. Another possibility would be backup jaws behind the leaves, under which leaf gaps could be parked. This could, however, introduce longer leaf travel paths and thereby increase treatment times and additionally require interdigitation capabilities. Furthermore, the capabilities of the backup jaw to reduce inter-leaf transmission vanishes for dynamic techniques like dMLC and VMAT as the backup jaws can only keep up with the most retracted leaf. Hence, leaves with a larger height can reduce the total transmission radiation. Concerning interdigitation, MLCs with such capabilities basically allow for independent placement of leaf positions for all leaf pairs. Generally, this facilitates more complex segment shapes as well as a larger search space of segment shapes for the sequencing algorithm to find an optimal plan. As example, interdigitation offers the possibility to treat multiple small field openings together within one segment, which may help to reduce the number of segments for a given degree of modulation of a treatment plan as compared to a non-interdigitating MLC. This potentially reduces the treatment time and MU, especially for larger and complex PTVs. The maximum speed of the leaves contributes to several properties. If the range of available speed is larger, faster plan delivery with a higher modulation degree is possible; also potentially reducing MU and scatter radiation.

**Table 1:** MLC parameters for MLCi2 and Agility-MLC

MLC parameter	MLCi2	Agility
Leaf width	10 mm	5 mm
Leaf speed	20 mm/s	65 mm/s
		incl. leaf guide
Min. leaf gap	5 mm	3 mm
Interdigitation	−/+	+
Backup jaws	yes	no

Thus, different parameters of the MLC can increase or reduce the degrees of freedom for the optimizer and deliverability to a different amount. The MLCi2 −/+ interdigitation and the Agility-MLC show differences in these parameters and capabilities (Table 1), and this study aims to investigate their contribution to different plans and treatment sites, which might be different and not easily foreseen *a priori*.

### Patients

Head and neck cases (HN), being considered challenging cases of current clinical practice [5], [27], and meningioma (MG) are chosen for the evaluation of the impact of MLC design and IMRT technique to the plan quality and potential benefits for the patient due to scatter radiation (MU) and treatment times. Each group includes five cases of previously treated patients. For HN, target volume delineation and dose prescription are according to the ACCRA study [28] requirements with two or three dose levels (61.6/50.4 Gy or 61.6/56/50.4 Gy), prescribed as SIB-technique in 28 fractions. MG were treated with 54 Gy in 30 fractions. The MG PTV includes the GTV based on MR and PET imaging with a margin of not more than 5 mm. Additional PRV-margins (3 to 5 mm) to critical structures (*e.g.* optical nerves) were applied to improve OAR sparing. Complete characteristics of the included datasets are shown in Tables 2 and 3.

**Table 2:** Patient characteristics

Meningioma	Location			PTV volume [cm^3^]	
WHO I					
MG1	os sphenoidale right			42	
M2G	frontobasal right, intra-/supra-sellar			107	
MG3	orbital/parasellar right, frontal			173	
MG4	temporal right			207	
MG5	parasellar			80

**Table 3:** Patient characteristics

HNSCC			PTV volume [cm^3^]		
Oropharynx	stage	location	PTV_61.6 Gy_	PTV_56 Gy_	PTV_50.4 Gy_
HN1	pT1 pN2b M0 L0 V0	right	132		834
HN2	pT2 pN2b M0 L1 V1	left	145	296	1240
HN3	pT3 pN2b M0 L1 V1	right	422	525	1006
HN4	pT2 pN2b M0 L0 V0	right	133	267	823
HN5	pT3 pN0 M0 L0 V0	left	166		659

### Optimization parameters

To access differences due to the IMRT-technique and the used MLC, all generated plans of the respective case are optimized applying the same patient-individual optimization parameters, *i.e.* same constrained optimization functions for OAR and PTV objectives with the related parameters, same gantry and collimator angles for ssIMRT and dMLC, as well as same Monte Carlo parameters (beam model of the radiation sources). Previous studies [29] show that VMAT plans generated using the Agility-MLC are superior in terms of higher PTV coverage and homogeneity without higher doses to organs at risk (OAR). Therefore, the applied optimization parameters are obtained from the VMAT^Agility^ plan separately for each case. To emphasize differences in the delivery of the techniques and the MLC properties, the applied constraints in this study are not those that were chosen for the clinical treatment plan generation. Instead, dose-limiting constraints are determined individually on a case-specific basis as low as realizable such that the PTV coverage is just not affected for the VMAT^Agility^ plan when using two or three full 360° arcs. These constraints are then used for all other plans of the respective case. Gantry angles for ssIMRT and dMLC are obtained from the ssIMRT^MLCi2-^ plan on the basis of the clinically used gantry angles.

Using constraint optimization, OARs will end up fulfilling the dose-limiting constraints (mainly) to the same extent and differences between the plans will be shown in terms of PTV coverage.

### Plan evaluation

Plans are evaluated using dose-volume-histogram (DVH) analysis and clinically important plan parameters. OAR exposure is evaluated based on QUANTEC criteria. For HN, D_max_ (dose to 1 % of the volume) for spinal cord, brain stem, plexus (brachialis ipsilateral, contralateral) and mandibula, D_mean_ and V_30Gy_ of the contralateral parotid gland as well as D_mean_ and V_50Gy_ of the larynx are evaluated. For MG, OARs are evaluated by means of D_max_ (dose to 1 % of the volume) for brainstem, chiasm, optic nerves, eyes, lenses and brain. Additionally, mean doses for the eyes and V_12Gy_ of the brain are evaluated.

PTV coverage analysis uses an equivalent uniform dose- (EUD-) definition based on the Poisson model [18], D_mean_, homogeneity (according to ICRU 83, formula 1), and conformity (suggested by Paddick [30], formula 2).

1 $$ H{I}_{ICRU}=\frac{D_{2\%}-{D}_{98\%}}{D_{50\%}} $$

The homogeneity index (HI) will tend to zero, the better the homogeneity of the plan is.

The conformity index (CI) accounts for prescribed dose outside the PTV and underdosage of the PTV, such that it equals 1, only if the prescription isodose is surrounding the PTV completely without extending into normal tissue. Otherwise CI is smaller. The CI is calculated for the 95 %- and 100 %-isodose:

2 $$ C{I}_{x\%}=\frac{{\left({V}_{D(PTV)=x\%}\right)}^2}{\left({V}_{PTV}\ast {V}_{D(body)=x\%}\right)} $$

Number of segments per plan, MU (as equivalent to the modulation degree) and estimated treatment times are compared as clinically important parameters.

As the PTV for each patient has quite a wide range in volume, location and proximity to OARs, the evaluation is done patient-wise. The following comparisons are made:

α) To have a general overview, each plan is compared to VMAT^Agility^.

β) In order to distinguish differences caused by the treatment technique independently of which MLC was used, each technique was compared to the corresponding VMAT plan using the same MLC. Therefore three underlying comparisons were made, which evaluate the influence of using sIMRT, dMLC or VMAT together with MLCi2^−^, MLCi2^+^ and Agility. In the results Tables 4 and 5, comparison of sIMRT and dMLC to VMAT using the MLCi2- would correspond to comparing column A/D *vs.* G (β^MLCi2-^), while comparing sIMRT and dMLC to VMAT using MLCi2+ and Agility corresponds to B/E *vs.* H (β^MLCi2+^) and C/F *vs.* I (β^Agility^), respectively.

**Table 4:** Results for nine plan groups (3 techniques + 3 MLC) for the evaluated parameters for the head-and-neck cases (average and standard deviation for five patients): QUANTEC criteria where applicable

Head and neck		Criteria	Step–and–shoot IMRT						dmlc IMRT						VMAT					
			MLCi2^−^		MLCi2^+^		Agility		MLCi2^−^		MLCi2^+^		Agility		MLCi2^−^		MLCi2^+^		Agility	
Segments^a^		–	97.6 ± 18.3	+	85.8 ± 14.0	+	**78.2 ± 11.7**		16930 ± 2.6	*=*	16935 ± 2.7	*=*	**171.2 ± 1.1**		338.0 ± 56.7	*=*	353.8 ± 76.6	*=*	**375.0 ± 57.5**	1
MU		–	86.1 ± 150.5	*=*	742.2 ± 109.3	*<*	657.2 ± 95.2	*<*	777.4 ± 65.8	*<*	803.4 ± 75.9	*<*	803.9 ± 87.1	*=*	959.5 ± 98.4	+	983.1 ± 123.1	+	**893.8 ± 96.3**	2
Estimated delivery time [s]		–	607.4 ± 92.0	+	571.1 ± 71.2	+	447.2 ± 55.7	+	421.2 ± 30.7	+	420.3 ± 25.0	+	323.2 ± 31.4	+	368.3 ± 28.6	+	361.7 ± 44.7	+	**224.4 ± 23.1**	3
PTV_50 4Gy_	EUD	50.4 ⇑	47.7 ± 0.2	*<*	47.7 ± 0.3	*<*	47.9 ± 0.5	*<*	48.8 ± 0.6	*<*	49.1 ± 0.6	*<*	49.0 ± 0.4	*<*	49.5 ± 0.8	*<*	49.5 ± 0.7	*<*	**50.1 ± 0.5**	4
	D_mean_	⇑	52.0 ± 1.6	*<*	52.1 ± 1.5	*<*	52.1 ± 1.5	*<*	52.9 ± 1.6	*<*	52.9 ± 1.6	*<*	53.0 ± 1.5	*<*	53.1 ± 1.8	*<*	53.1 ± 1.7	*<*	**53.3 ± 1.7**	5
	HI_ICRU_	⇓	0.436 ± 0.041	*+*	0.435 ± 0.041	*+*	0.431 ± 0.037	*+*	0.408 ± 0.031	*+*	0.399 ± 0.032	*+*	0.404 ± 0.036	*+*	0.380 ± 0.032	*+*	0.382 ± 0.031	*+*	**0.373 ± 0.032**	6
	CI_100 %_	⇑	0.420 ± 0.098	*<*	0.433 ± 0.090	*<*	0.429 ± 0.088	*<*	0.495 ± 0.075	*<*	0.507 ± 0.071	*<*	0.522 ± 0.066	*=*	0.523 ± 0.106	*<*	0.521 ± 0.088	*<*	**0.566 ± 0.091**	7
	CI_95 %_	⇑	0.617 ± 0.040	*<*	0.629 ± 0.038	*<*	0.634 ± 0.031	*<*	0.678 ± 0.027	*=*	0.684 ± 0.028	*=*	0689 ± 0.026	*=*	0.692 ± 0.034	*<*	0.691 ± 0.031	*<*	**0.711 ± 0.033**	8
PTV_61.6 Gy_	EUD	61.6 ⇑	58.4 ± 1.2	*<*	58.6 ± 1.0	*<*	58.9 ± 1.3	*<*	59.9 ± 0.8	*<*	60.1 ± 0.9	*<*	60.3 ± 0.9	*=*	60.3 ± 1.0	*<*	60.2 ± 1.0	*<*	**60.7 ± 0.9**	9
	D_mean_	⇑	60.3 ± 0.7	*<*	60.4 ± 0.7	*<*	60.5 ± 0.8	*<*	61.2 ± 0.5	*<*	61.3 ± 0.6	*<*	61.4 ± 0.6	*=*	61.4 ± 0.7	*<*	61.3 ± 0.7	*<*	**61.6 ± 0.6**	10
	HI_ICRU_	⇓	0.175 ± 0.035	*+*	0.172 ± 0.036	*+*	0.165 ± 0.040	*+*	0.140 ± 0.028	*+*	0.135 ± 0.030	*+*	0.127 ± 0.033	*=*	0.128 ± 0.025	+	0.132 ± 0.028	*+*	**0.120 ± 0.027**	11
	CI_100 %_	⇑	0.322 ± 0.084	*<*	0.326 ± 0.098	*<*	0351 ± 0.136	*<*	0.476 ± 0.113	*<*	0.501 ± 0.127	*<*	0.539 ± 0.133	*=*	0.517 ± 0.149	*<*	0.502 ± 0.132	*<*	**0580 ± 0.146**	12
	CI_95 %_	⇑	0.702 ± 0.04	*<*	0.704 ± 0.032	*<*	0.725 ± 0.048	*=*	0.775 ± 0.033	*=*	0.776 ± 0.033	*=*	0.779 ± 0.032	*=*	0.782 ± 0.037	*=*	0.779 ± 0.036	*=*	**0.787 ± 0.037**	13
Spinal cord	D_max_	50	35.9 ± 4.5	*=*	35.4 ± 4.0	*=*	35.1 ± 3.9	*=*	36.5 ± 4.9	*=*	35.5 ± 4.0	*=*	35.6 ± 4.1	*=*	36.0 ± 4.1	*=*	35.7 ± 4.5	*=*	**35.5 ± 3.9**	14
Brainstem		52	40.1 ± 2.1	*=*	39.7 ± 2.1	*=*	39.8 ± 2.4	*=*	39.0 ± 2.9	*=*	39.6 ± 2.4	*=*	40.5 ± 2.4	*=*	40.0 ± 3.0	*=*	39.1 ± 2.8	*=*	**39.7 ± 2.7**	15
Plexus ipsilateral		61.6 ⇓	58.5 ± 5.0	*=*	58.6 ± 5.6	*=*	58.6 ± 5.6	*=*	59.0 ± 5.1	*=*	59.0 ± 5.2	*=*	59.0 ± 5.1	*=*	59.0 ± 5.1	*=*	59.0 ± 5.2	*=*	**58.9 ± 5.1**	16
Plexus contralateral		50.4 ⇓	51.3 ± 1.1	*=*	51.0 ± 0.7	*=*	50.8 ± 1.4	*=*	50.9 ± 0.5	*=*	51.1 ± 0.5	*=*	51.2 ± 0.7	*=*	51.5 ± 1.0	*=*	51.5 ± 0.9	*=*	**51.3 ± 0.8**	17
Mandibula		61.6 ⇓	60.3 ± 3.5	*=*	60.5 ± 3.6	*=*	60.4 ± 2.6	*=*	60.8 ± 2.3	*=*	60.1 ± 1.9	*=*	61.1 ± 1.9	*=*	61.4 ± 2.3	*=*	61.1 ± 2.7	*=*	**61.6 ± 2.4**	18
Parotid contralateral	D_mean_	26	23.9 ± 1.7	*<*	23.6 ± 2.3	*<*	23.8 ± 0.8	*<*	25.3 ± 2.2	*=*	25.6 ± 1.7	*=*	25.1 ± 1.6	*=*	24.8 ± 1.9	*=*	24.8 ± 1.8	*=*	**25.1 ± 1.7**	19
	V_30 Gy_	50	34.2 ± 6.3	*=*	33.6 ± 7.4	*=*	33.9 ± 4.5	*=*	36.3 ± 6.4	*=*	37.9 ± 5.8	*+*	36.7 ± 5.4	*+*	33.8 ± 6.8	*=*	34.3 ± 6.0	*=*	**34.9 ± 6.0**	20
Larynx	D_mean_	⇓	46.7 ± 1.2	*<*	46.3 ± 1.5	*<*	47.2 ± 1.0	*=*	47.2 ± 1.1	*=*	47.3 ± 0.9	*=*	47.7 ± 0.9	*=*	47.4 ± 1.8	*=*	47.2 ± 1.6	*=*	**47.8 ± 1.5**	21
	V_50 Gy_	⇓	14.0 ± 12.3	*=*	13.9 ± 12.2	*=*	14.9 ± 15.2	*=*	16.7 ± 13.5	*=*	17.7 ± 14.4	*=*	19.3 ± 14.6	*=*	18.4 ± 15.4	*=*	17.5 ± 18.0	*=*	**21.0 ± 18.9**	22
			A		B		C		D		E		F		G		H		I	

Arrows indicate if a higher or a lower value gives the better plan. “<”,”+” or “=” indicate whether the result is lower, higher or equal to the VMAT^Agilty^ according to comparison α (bold) result on a significance level of 5 %, ^a^ for segments: results are compared within each technique to the respective Agility plan according to comparison γ (bold)

**Table 5:** Results for nine plan groups (3 techniques + 3 MLC) for the evaluated parameters for the meningioma cases (average and standard deviation for five patients): QUANTEC criteria where applicable

Meningioma		Criteria	Step–and–shoot IMRT						dmlc IMRT						VMAT					
			MLCi2^−^		MLCi2^+^		Agility		MLCi2^−^		MLCi2^+^		Agility		MLCi2^−^		MLCi2^+^		Agility	
Segments^a^		–	35.8 ± 10.9	=	36.6 ± 11.5	=	**33.8 ± 11.3**		158.8 ± 27.5	=	159.4 ± 28.9	=	**158.4 ± 24.9**		250.4 ± 26.2	=	259.8 ± 25.3	=	**26638 ± 28.1**	1
MU		–	480.8 ± 140.9	=	474.4 ± 128.8	<	452.7 ± 124.9	<	577.9 ± 166.3	=	609.3 ± 194.4	=	612.5 ± 175.5	=	625.4 ± 125.4	=	642.0 ± 143.5	=	**569.0 ± 95.5**	2
Estimated delivery time [s]		–	290. ± 90.8	+	297.5 ± 92.3	+	242.1 ± 69.9	+	300.9 ± 91.7	+	299.5 ± 101.9	+	252.4 ± 90.5	+	209.4 ± 24.1	+	209.0 ± 26.7	+	**132.3 ± 9.1**	3
PTV	EUD	54 ⇑	50.8 ± 2.3	<	51.1 ± 1.9	<	50.9 ± 2.2	<	53.2 ± 0.2	<	53.2 ± 0.3	<	53.5 ± 0.3	<	53.3 ± 0.6	<	53.5 ± 0.1	<	**53.8 ± 0.2**	4
	D_mean_	54⇑	52.6 ± 0.6	<	52.9 ± 0.5	<	52.9 ± 0.5	<	53.9 ± 0.2	<	53.9 ± 0.2	<	54.2 ± 0.2	<	54.0 ± 0.4	<	54.1 ± 0.1	<	**54.3 ± 0.2**	5
	HI_ICRU_	⇓	0.198 ± 0.072	+	0.187 ± 0.073	+	0.198 ± 0.063	+	0.142 ± 0.043	+	0.139 ± 0.045	+	0.124 ± 0.041	+	0.133 ± 0.045	+	0.125 ± 0.027	+	**0.111 ± 0.034**	6
	CI_100 %_	⇑	0.267 ± 0.056	<	0.284 ± 0.056	<	0.323 ± 0.066	<	0.499 ± 0.025	<	0.492 ± 0.027	<	0.590 ± 0.028	<	0.523 ± 0.044	<	0.539 ± 0.045	<	**0.638 ± 0.021**	7
	CI_95 %_	⇑	0.622 ± 0.117	<	0.647 ± 0.102	<	0.647 ± 0.107	<	0.750 ± 0.059	=	0.747 ± 0.057	=	0.766 ± 0.062	=	0.760 ± 0.044	=	0.759 ± 0.057	=	**0.768 ± 0.055**	8
Brainstem	D_max_	54	47.9 ± 10.3	=	48.2 ± 10.4	=	48.2 ± 10.1	=	48.1 ± 10.2	=	48.2 ± 10.3	=	48.3 ± 10.6	=	48.1 ± 10.2	=	48.1 = 10.2	=	**48.3 ± 10.1**	9
Chiasm		54	53.3 ± 0.7	=	53.5 ± 0.4	=	53.8 ± 0.3	=	53.6 ± 0.4	=	53.7 ± 0.3	=	53.7 ± 0.3	=	53.8 ± 0.3	=	53.8 = 0.2	=	**53.8 ± 0.3**	10
Opt. nerve left		54	41.7 ± 15.9	=	42.0 ± 16.0	=	42.1 ± 16.0	=	42.1 ± 16.1	=	42.1 ± 16.0	=	42.1 ± 16.1	+	42.1 ± 15.8	=	42.0 = 16.1	=	**41.9 ± 16.2**	11
Opt. nerve right		54	53.7 ± 0.6	=	53.9 ± 0.2	=	53.8 ± 0.3	=	53.8 ± 0.1	_=_	53.9 ± 0.1	=	53.8 ± 0.1	=	53.8 ± 0.0	=	53.9 = 0.1	=	**53.9 ± 0.1**	12
Lens left		⇓	4.0 ± 1.3	<	4.1 ± 1.2	<	4.1 ± 1.1	<	4.9 ± 1.0	=	4.7 ± 1.3	=	5.0 ± 1.2	=	5.9 ± 0.3	=	6.1 = 0.0	=	**6.0 ± 0.1**	13
Lens right		⇓	11.1 ± 9.5	=	11.3 ± 10.0	=	11.1 ± 8.8	=	16.0 ± 10.8	=	14.9 ± 10.3	=	14.4 ± 9.8	=	16.7 ± 11.8	=	16.9 = 11.4	=	**16.4 ± 10.2**	14
Bulb left	D_mean_	⇓	7.9 ± 2.4	=	7.7 ± 2.2	=	7.5 ± 1.7	<	8.4 ± 2.0	=	8.3 ± 2.1	=	8.5 ± 2.2	=	8.9 ± 1.7	=	9.1 = 1.5	=	**9.1 ± 1.5**	15
	D_max_	54	20.0 ± 5.2	=	19.8 ± 5.3	=	20.2 ± 4.5	=	20.5 ± 5.6	=	20.1 ± 4.9	=	21.5 ± 5.6	=	19.3 ± 8.7	=	19.7 ± 8.4	=	**20.1 ± 8.0**	16
Bulb right	D_mean_	⇓	19.0 ± 7.9	=	19.1 ± 8.0	=	20.1 ± 8.4	=	22.5 ± 10.7	=	21.9 ± 10.3	=	22.4 ± 10.9	=	23.0 ± 11.1	=	23.4 ± 11.4	=	**23.2 ± 10.9**	17
	D_max_	54	40.1 ± 13.4	=	39.7 ± 13.3	=	40.5 ± 12.7	=	41.4 ± 13.9	=	41.5 ± 15.0	=	41.3 ± 14.3	=	41.2 ± 13.9	=	41.1 ± 14.6	=	**41.0 ± 14.5**	18
Brain	D_max_	54	53.9 ± 0.8	<	54.1 ± 0.8	<	54.1 ± 0.9	<	54.6 ± 0.6	<	54.5 ± 0.7	<	54.8 ± 0.7	=	54.7 ± 0.8	<	54.8 ± 0.6	<	**54.9 ± 0.6**	19
	V_12 Gy_	⇓	43.6 ± 16.4	=	43.7 ± 16.4	=	43.4 ± 15.6	=	43.3 ± 15.7	=	43.6 ± 16.1	=	42.5 ± 16.0	=	43.8 ± 14.8	=	44.6 ± 14.6	=	**43.5 ± 13.9**	20
			A		B		C		D		E		F		G		H		I	

Arrows indicate if a higher or a lower value gives the better plan. “<”,”+” or “=” indicate whether the result is lower, higher or equal to the VMAT^Agilty^ according to comparison α (bold) result on a significance level of 5 %, ^a^ for segments: results are compared within each technique to the respective Agility plan according to comparison γ (bold)

γ) In order to distinguish differences caused by MLC independently of which technique was used, each MLC was compared to the corresponding Agility plan using the same technique. Therefore three underlying comparisons were made, which evaluate the influence of using MLCi2^−^, MLCi2^+^ or Agility together with sIMRT, dMLC and VMAT. In the results Tables 4 and 5, comparison of MLCi2^−/+^ to Agility for ssIMRT would correspond to comparing column A/B *vs.* C (γ^ssIMRT^), while comparing MLCi2^−/+^ to Agility for dMLC or VMAT corresponds to D/E *vs.* F (γ^dMLC^) and G/H *vs.* I (γ^VMAT^), respectively.

For each of the five patients per group, nine plans combining three different IMRT-techniques (ssIMRT, dMLC, VMAT) and three different MLCs (MLCi2 −/+ interdigitation, Agility-MLC) are generated. Thereby, plans are distinguished not only by MLC or technique but both – MLC and technique.

To conclude the results of the presented study, the mean and standard deviation for each evaluated parameter over the 5 patients per group as well as the results of the paired *T*-test (significance level: 0.05) for comparisons α, β and γ are calculated. For a general overview, Tables 4 and 5 show the results for comparison α (comparison γ for segments).

## Results

In this study, 10 patients (five HN, five MG) are optimized for ssIMRT (columns A-C, Tables 4 and 5), dMLC (columns D-F, Tables 4 and 5) and VMAT (columns G-I, Tables 4 and 5). For each technique, the MLCi2-MLC without (−) (columns A/D/G, Tables 4 and 5) and with (+) (columns B/E/H, Tables 4 and 5) interdigitation and the Agility-MLC (columns C/F/I, Tables 4 and 5) are used. Thereby, 9 plans per patient are generated. The VMAT^Agility^ plan is considered as a reference for comparisons α, as this is the plan used to determine all optimization parameters. An overview of all results of the head-and-neck and meningioma cases is shown in Tables 4 and 5, respectively. These tables include the results of a paired *T*-test, but only for comparison α with the VMAT^Agility^ plans. If the plan compared to is another one, results of the paired *T*-test are found in the text. For better understanding, indices from Tables 4 and 5 are used anyway.

As constrained optimization is used, dose to the OAR differed only slightly and all QUANTEC criteria are met. If OAR have to be spared more, the cost would be less PTV coverage. Among all plans, VMAT^Agility^ show best coverage and fastest delivery. Comparing different techniques (β), VMAT always shows the fastest delivery. Comparing MLCs (γ), Agility always shows the fastest delivery. Least MU are found for ssIMRT plans using the Agility-MLC. The total amount of MU depends on the PTV size and complexity as well as on the used technique. The total number of segments is independent of the used MLC for MG cases as well as dMLC and VMAT for HN cases.

**Fig. 1:**
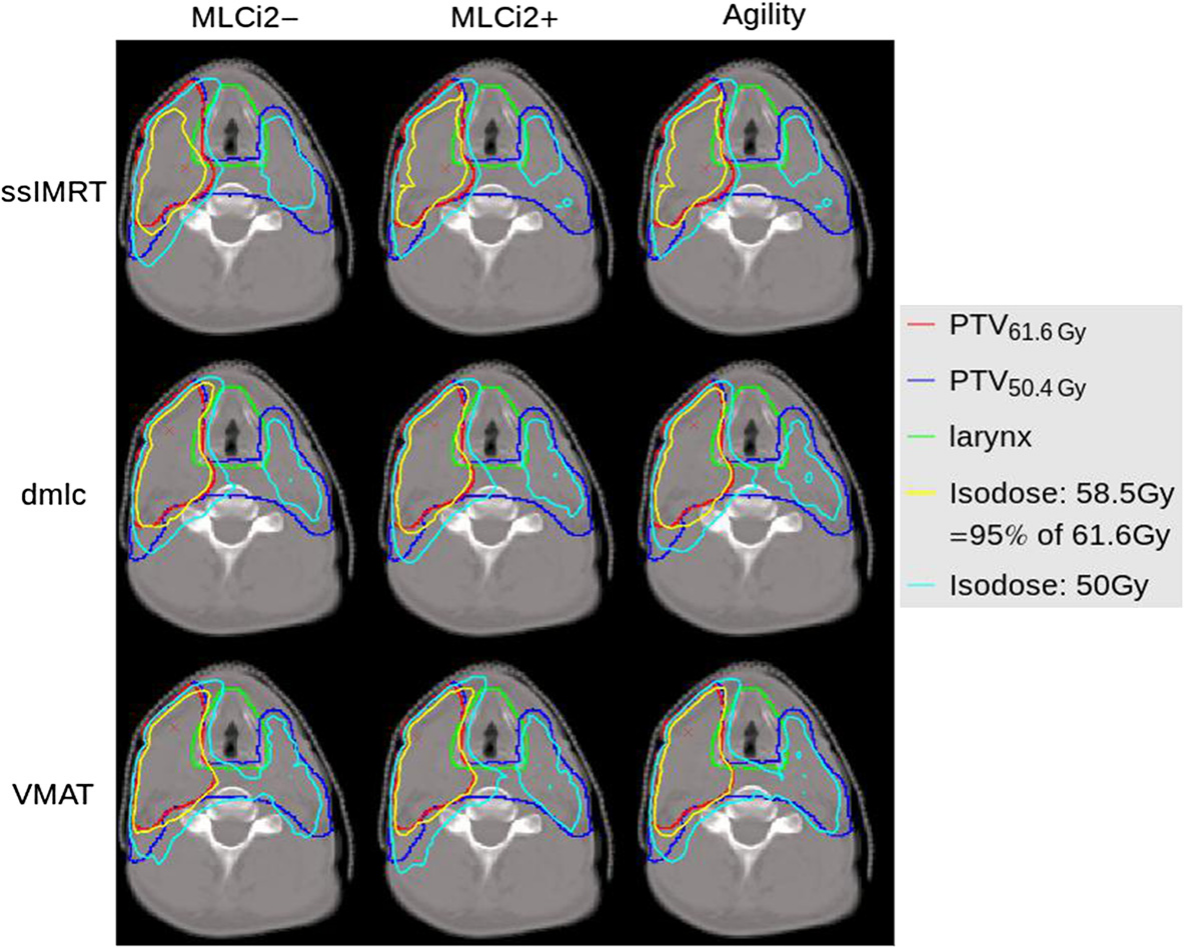
By example of patient case HN3, relevant isodoses for the larynx (50Gy, 58.5Gy) are shown for the nine generated plans using ssIMRT, dMLC and VMAT with MLCi2 without (−) and with (+) interdigitation and the Agility-MLC
  For nerve structures (spinal cord – A-I 14, Table 4, brainstem – A-I 15, Table 4, plexus – A-I 16 + 17, Table 4) differences in D_max_ between techniques and MLCs are not statistically different from the VMAT^Agility^ plan according to the paired *T*-test and not larger than 1.5Gy. Within ssIMRT (γ^ssIMRT^, A-C, Table 4), Agility plans (C 14–17, Table 4) have equal or lower D_max_. Also D_max_(mandibula) (α, A-I 18, Table 4) overall does not deviate more than 1.5Gy, but with lower doses in general for all ssIMRT plans (ΔD_max_ > 1.0Gy, A-C 18, Table 4).
  The contralateral parotids (A-I 19 + 20, Table 4) have the lowest D_mean_ in ssIMRT (ΔD_mean_ > 1.2Gy, p ≤ 0.02, A-C 19, Table 4). Evaluating V_30Gy_, dMLC^MLCi2+^ and dmlc^Agility^ plans show V_30Gy_ elevated at least by 5 % (E + F 20, p < 0.01, Table 4). Comparing MLCs within each technique (γ), significant (p ≤ 0.02, Table 4) differences are only found for the comparison dmlc^MLCi2+^*vs.* dMLC^Agility^ (E 19 + 20 *vs.* F 19 + 20, Table 4), showing higher D_mean_ and V_30Gy_ for dmlc^MLCi2+^.
- *Head-and-Neck – PTV coverage*
  All OAR sparing compared to VMAT^Agility^ comes at the cost of less PTV coverage. For all criteria (EUD, D_mean_, HI, CI_100 %_, CI_95 %_ / A 4-I 13, Table 4), VMAT^Agility^ (I 4–13, Table 4) has the best PTV coverage.
  For PTV_50.4Gy_ (A 4-I 8, Table 4), dMLC plans have at least 1.0Gy (p ≤ 0.01) and 0.4Gy (p ≤ 0.01) less EUD (D-F 4, Table 4) and D_mean_ (D-F 5, Table 4), respectively, and ssIMRT plans have at least 2.1Gy (p ≤ 0.01) and 1.2Gy (p≤0.01) less EUD (A-C 4, Table 4) and D_mean_ (A-C 5, Table 4), respectively. For CI_95 %_(PTV_50.4Gy_), all dmlc plan show no significant difference compared to the VMAT^Agility^ plan (D-E 8 *vs.* I 8, Table 4). Evaluating the coverage of PTV_61.6Gy_, the dMLC^Agility^ plans show no significant differences in the evaluated parameters (F 9–13 *vs.* I 9–13, Table 4). For PTV_61.6Gy_ only ssIMRT^MLCi2-/+^ have significant less conformity of the 95 %-isodose (A + B 13 *vs.* I 13, Table 4).
  Comparing different MLCs within the techniques (γ: A + B *vs.* C, D + E *vs.* F, G + H *vs.* I, Table 4), significant differences for the PTV coverage in ssIMRT (γ^ssIMRT^: A + B 4–13 *vs.* C 4–13, Table 4) are found for CI_95 %_ (PTV_50.4Gy_) (p ≤ 0.01) (A 8 *vs.* C 8, Table 4) and EUD(PTV_61.6Gy_) (p ≤ 0.01) (A 9 *vs.* C 9, Table 4). Within the dMLC plans(γ^dMLC^), MLCi2^+^ shows significant differences only for PTV_61.6Gy_ (D_mean_, HI and CI_100 %_, E 10–12 *vs.* F 10–12, Table 4), while MLCi2^-^ has less PTV coverage (p = 0.01) in terms of D_mean_ (-1.2Gy) and CI_100 %_ (-5 %) for PTV_50.4Gy_ and in terms of EUD (-0.4Gy), D_mean_ (-0.2Gy), HI (-10 %) and CI_100 %_ (-12 %) for PTV_61.6Gy_ (D 4–13 *vs.* F 4–13, Table 4).
  Comparing different techniques using the same MLC (ß: A + D *vs.* G, B + E *vs.* H, C + F *vs.* I, Table 4), ssIMRT plans have worse PTV coverage (p ≤ 0.01) using either of the three MLCs. Using MLCi2^-^ (ß^MLCi2-^), dMLC plans show less homogeneity (p ≤ 0.02) for PTV_50.4Gy_ and PTV_61.6Gy_ and less EUD (-0.4Gy) for PTV61.6Gy compared to VMAT^MLCi2-^. For dMLC^MLCi2+^ (ß^MLCi2+^), significant (p ≤ 0.01) differences for the PTV coverage compared to VMAT^MLCi2+^ are only found for HI(PTV_50.4Gy_) (-4 %) (E 4–13 *vs.* H 4–13, Table 4), whereas the PTV coverage for dMLC using the Agility-MLC (ß^Agility^) is less in terms of EUD, D_mean_, HI and CI_100 %_ for both PTVs compared to VMAT^Agility^ (F 4–13 *vs.* I 4–13, Table 4).
  Figure 2 shows the results for one HN patient (HN3 in Tables 3) as an example. Figure 2d depicts that the evaluated OAR parameters differ only slightly between the techniques and used MLC. As mentioned, larger differences are found for V_50Gy_(larynx) (A-I 22, Table 4), D_max_(mandibula) (A-I 18, Table 4) and the contralateral parotid (D_mean_, V_30Gy_) (A-I 19 + 20, Table 4). In contrast to the only slightly differing clinically important OAR parameters, Fig. 2a, b and c depict the differences of the PTV coverage: HI, CI and D_mean_ drop clearly, if ssIMRT is used. Influences of the MLC design are only small and highest in combination with VMAT.

Detailed results are as follows:

- *Head-and-Neck – OAR exposure*
  As constrained optimization is used, all OARs of the respective case fulfill the prescribed dose-limiting constraints. These constraints are optimized case-specific for this study, based on the VMAT^Agility^ plan (I1-I22, Table 4), and are not the ones clinically chosen, QUANTEC criteria are met for all OAR except for the mean dose of the larynx (A-I 21, Table 4).
  This is because the PTV_50.4Gy_ surrounds the larynx in all HN cases, an example case (HN3) is shown in Fig. 1. Thereby, the QUANTEC criteria of D_mean_ < 44Gy (A-I 21, Table 4) is barely achievable, if the PTV is expected to receive the full dose. Due to the irradiation from all gantry angles, D_mean_ (A-I 21, Table 4) and V_50Gy_ (A-I 22, Table 4) for the larynx are always highest for VMAT, even though significant differences (p ≤ 0.01) are only found for ssIMRT^MLCi2−/+^ (D_mean_, A + B 21, Table 4).

**Fig. 2:**
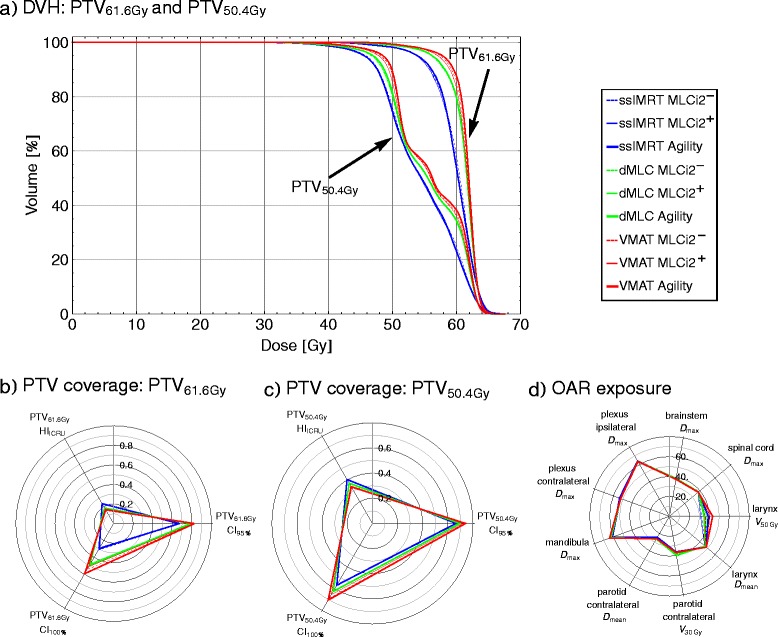
Example of one head-and-neck case (HN3). **a** DVHs for PTV_61.6Gy_ and PTV_50.4Gy_ of all nine generated plans, (**b**) homogeneity and conformity indices for PTV_61.6Gy_ and **c**) PTV_50.4Gy_, **d**) evaluated QUANTEC and clinical criteria for OARs
- *Meningioma – OAR exposure*
  As for the HN cases, all generated plans fulfill the prescribed constraints and predefined criteria. Larger standard deviations for the brainstem (A-I 9, Table 5), left optical nerve (A-I 11, Table 5), right lens (A-I 14, Table 5) and bulb (A-I 17 + 18, Table 5) are found due different proximity of the PTV to these OARs for the different patients (α).
  Differences between the techniques and MLCs are found for the eyes (A-I 13–18, Table 5). Among all plans (α), ssIMRT plans preserve lenses and bulbs best (ΔD_mean_(bulbs) ≤ 4.2Gy, p ≥ 0.05 (A-C 15/17, Table 5), ΔD_max_(lenses) ≤ 5.3Gy, p ≤ 0.02 (A-C 13 + 14, Table 5)), while dMLC plans preserve the lenses (ΔD_max_ = 0.4 - 2.0Gy, p ≥ 0.07 (D-F 13 + 14, Table 5)) and the bulbs (ΔD_mean_ = 0.6 - 1.4Gy, p ≥ 0.06 (D-F 15/17, Table 5)) better than VMAT. Reasons are (1) the use of the advanced segment shape optimization algorithm for VMAT during the second optimization step that enables to fully take advantage of the constraints by placing the leaves more effectively with respect to PTV coverage and therefore nearer to the OAR, and (2) VMAT uses the full range of possible gantry angles, while the gantry angles for ssIMRT and dMLC were chosen, such that they would avoid radiation to the eyes more effectively. The use of Agility shows an influence when using dMLC (γ^dMLC^, F *vs.* D + E, Table 5), presumably due to its interdigitation capabilities and smaller leaves. Overall, only the differences for the left lens for the ssIMRT plans are significant when comparing to VMAT^Agility^.
  Furthermore, significantly (p ≤ 0.01) lower D_max_ (upto 1Gy) are found for brain (A-I 20, Table 5). Only for dMLC^Agility^ and VMAT^MLCi2-^, these changes are significant (p ≤ 0.05). The maximal dose of the brain lies within the PTV. As the PTV coverage is best for VMAT^Agility^ (see next paragraph), higher D_max_(brain) of these plans can be explained by this.
- *Meningioma – PTV coverage*
  As for the HN cases, PTV coverage for the meningioma cases is best for VMAT^Agility^ (A-I 4–8, Table 5). EUD, D_mean_, HI and CI_100 %_ show less PTV coverage for all other plans. Differences for CI_95 %_ are non-significant for dMLC and VMAT using either MLC (D-I 8, Table 5). Larger differences for the PTV coverage (ΔEUD ≥ 2.7Gy (p ≤ 0.02), ΔD_mean_ ≥ 1.4Gy (p ≤ 0.02)) are found for all ssIMRT plans (A-C 4–8 *vs.* I 4–8), showing that also for meningioma, the technique is more essential than the MLC design. For all ssIMRT, evaluated parameters show much less PTV coverage than dMLC or VMAT (A-C 4–8 *vs.* D-I 4–8, Table 5).
  Comparing MLCs within the used techniques (γ: A + B *vs.* C, D + E *vs.* F, G + H *vs.* I, Table 5), the Agility-MLC shows significantly higher PTV coverage for the conformity (p ≤ 0.02) of ssIMRT (γ^ssIMRT^), but also for EUD, D_mean_, HI und CI_100 %_ within dMLC (γ^dMLC^) or VMAT plans (γ^VMAT^) (p ≤ 0.01 (dMLC), p = 0.02 (VMAT)). Therefore, the use of the Agility-MLC has more impact for dMLC and VMAT.
  Comparing different techniques using the same MLC (ß: A + D *vs.* G, B + E *vs.* H, C + F *vs.* I, Table 5), MLCi2^-^ has (p ≤ 0.01) less PTV coverage for ssIMRT (ß^MLCi2-^). Using the MLCi2 with interdigitation, differences within dMLC plans become significant (p ≤ 0.02) for EUD, D_mean_ and CI_100 %_ (ß^MLCi2+^).
  Even though not significant (p ≥ 0.07), dMLC^Agility^ show better homogeneity and conformity than any VMAT^MLCi2^ (F 6–8 *vs.* H 6–8, Table 5), showing that (1) the used gantry angles are chosen such, that comparable plans to VMAT^Agility^ are possible and (2) smaller leaves are favorable when small OAR within the PTV need to be preserved. Still the PTV coverage in terms of EUD and D_mean_ for dMLC (all MLCs) are within the range of the VMAT plans (ΔEUD = 0.2 - 0.6Gy (D-F 4 *vs.* G-I 4, Table 5), ΔD_mean_ = 0.2 - 0.4Gy (D-F 5 *vs.* G-I 5, Table 5)). For all ssIMRT, evaluated parameters show much less PTV coverage than dMLC or VMAT (A-C 4–8 *vs.* D-I 4–8, Table 5).
  Figure 3 shows the results for one meningioma patient (MG2 in Tables 2) as an example. Firgure 3c depicts that the evaluated OAR parameter differ only for the bulbs (D_mean_, D_max_) and the lenses (D_max_). In contrast, Fig. 3a and b depict the differences of the PTV coverage: homogeneity, conformity and D_mean_ drop clearly, if ssIMRT is used. Influences of the MLC design are only small and highest in combination with VMAT.

**Fig. 3:**
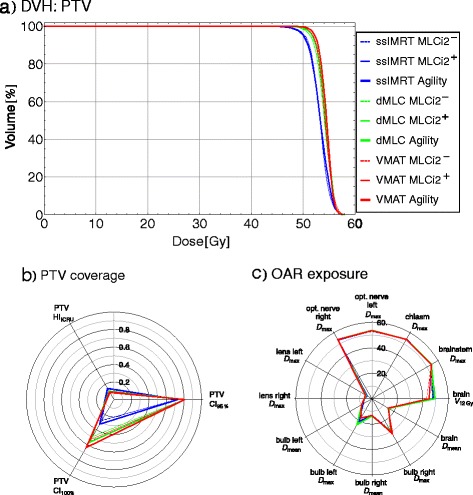
Example of one meningioma case (MG2). **a** DVH for the PTV of all nine generated plans, (**b**) homogeneity and conformity indices for the PTV, c) evaluated QUANTEC and clinical criteria for OARs
- *Estimated treatment times*
  The reported treatment times are not measured, but estimated from the TPS with realistic assumptions for the dose rate, leaf and gantry rotation velocity. Hence, times within this study are at least comparable among each other. As absolute times depend on the maximal achievable dose rate, leaf and gantry rotation velocity of a specific linac, the actual delivery times may deviate about few percent from those treatment times estimates. However, differences between techniques or MLCs from the presented study may be compared to other studies in a relative manner.
  The analysis of the estimated treatment time shows, that the combination of VMAT and Agility results in the fastest treatment for each case. This is still true when using up to three arcs for HN cases and the time difference can be up to 481 s (≈8 min) for single cases (HN2), comparing VMAT^Agility^*vs.* ssIMRT^MLCi2-^.
  Comparing each technique using either MLC (ß: A + D 3 *vs.* G 3, B + E 3 *vs.* H 3, C + F 3 *vs.* I 3, Tables 4 and 5), fastest (p ≤ 0.01) treatment plans are found among VMAT, regardless of the PTV complexity (shape, dose levels, proximity to OARs), while ssIMRT takes the most time (A-C 3 *vs.* D-F 3 *vs.* G-I 3, Tables 4 and 5). The MLC influences the amount of time that can be reduced by changing from one technique to another. VMAT^MLCi2-/+^ reduces the treatment time about 60 % compared to ssIMRT with MLCi2^-/+^ (ß^MLCi2-/+^: A + B 3 *vs.* G + H 3, Tables 4 and 5) and almost 50 % with Agility (ß^Agility^: C 3 *vs.* I 3, Tables 4 and 5). For dMLC, treatment times are reduced by about 15 % (ß^MLCi2-/+^: D + E 3 *vs.* G + H 3, Tables 4 and 5) and 45 % (ß^Agility^: F 3 *vs.* I 3, Tables 4 and 5) using MLCi2^-/+^ and Agility, respectively.
  Comparing the MLCs using either technique (γ), Agility on average offers faster (p ≤ 0.01) treatments by 48/55 s for ssIMRT (γ^ssIMRT^: A + B 3 *vs.* C 3, Table 5), 48/47 s for dMLC (γ^dMLC^: D + E 3 *vs.* F 3, Table 5) and 77/76 s for VMAT (γ^VMAT^: G + H 3 *vs.* I 3, Table 5) for meningioma using the MLCi2^-/+^. This corresponds to about 20 % longer treatments for ssIMRT and dMLC and 58 % longer treatments for VMAT with MLCi2. For the larger and more complex PTVs of the HN cases, Agility is faster by 160/124 s for ssIMRT(γ^ssIMRT^: A + B 3 *vs.* C 3, Table 4), 98/97 s for dMLC (γ^dMLC^: D + E 3 *vs.* F 3, Table 4) and 143/137 s for VMAT (γ^VMAT^: G + H 3 *vs.* I 3, Table 4) for MLCi2^-^ and MLCi2^+^, respectively, corresponding to 28 and 36 % longer treatments for ssIMRT, 30 % longer treatments for dMLC and over 60 % longer treatments for VMAT when using the MLCi2.
  The ability of interdigitation of the MLCi2 does not reduce treatment times much. Larger time reduction is found for ssIMRT, only (HN: 36 s, 10 %, p = 0.07, A 3 *vs.* B 3, Table 4, MG: 7 s, 3 %, p ≤ 0.01, A 3 *vs.* B 3, Table 5).
- *Monitor units*
  MU for HN and MG are least for ssIMRT^MLCi2+^ and ssIMRT^Agility^ (B/C 2, Tables 4 and 5).
  Compared to MLCi2^-^ and MLCi2^+^, Agility saves MU for HN cases using ssIMRT (32 %, 13 %, p ≤ 0.01 (γ^ssIMRT^: A/B 2 *vs.* C 2, Table 4)) and VMAT (7 %, 10 %, p ≤ 0.01 (γ^VMAT^: G/H 2 *vs.* I 2, Table 4)), but not for dMLC (γ^dMLC^: D/E 2 *vs.* F 2, Tables 4 and 5) or MG cases (γ: A/B 2 *vs.* C 2, D/E 2 *vs.* F 2, G/H 2 *vs.* I 2, Table 5).
  Comparing each technique using either MLC for MG cases (ß), ssIMRT plans have the least MU as compared to the respective VMAT plans (ΔMU = 23 %/26 %/20 %, p ≤ 0.01, MLCi2^-^/MLCi2^+^/Agility, A/B/C 2 *vs.* G/H/I 2, Table 5), while no significant changes (p ≥ 0.36) between dMLC and VMAT are found. For HN cases VMAT increases MU compared to dMLC (ΔMU = 19 %/18 %/10 %, p ≤ 0.01, MLCi2^-^/MLCi2^+^/Agility, D/E/F 2 *vs.* G/H/I 2, Table 4) and ssIMRT (ΔMU = 9 %/25 %/26 %, p ≤ 0.01, MLCi2^-^/MLCi2^+^/Agility, A/B/C 2 *vs.* G/H/I 2, Table 4). Interdigitation reduces the MU for HN cases for ssIMRT by 15 % (p ≤ 0.01) (A 2 *vs.* B 2, Table 4).
  This shows that the reduction of MU depends not only on the size and complexity of the treated volume but also on the technique and the MLC.
- *Segments*
  The number of segments for the different MLCs for a given treatment technique (γ) is also investigated (A-I 1, Tables 4 and 5).
  For dMLC and VMAT (γ^dMLC/VMAT^), the amount of segments does not change significantly with the MLC and only slightly with the plan complexity (mean number of segments: MG: 159 (dMLC, D-F 1, Table 5)/259 (VMAT, G-I 1, Table 5), HN: 170 (D-F 1, Table 4)/356 (VMAT, G-I 1, Table 4)).
  For ssIMRT (γ^ssIMRT^), segments do not change with the MLC (34–37 segments, p ≥ 0.15 (A-C 1, Table 5)) for the MG cases, but for the HN cases (+19/8 segments MLCi2^-/+^, p ≤ 0.01,(A-C 1, Table 4)). Interdigitation reduces the amount of segments by 12 % (p ≤ 0.01) for HN cases using ssIMRT (A 1 *vs.* B 1, Table 4).

## Discussion

This work evaluates the impact of the MLC design (MLCi2 without (−) and with (+) interdigitation, and Agility-MLC) with regard to different IMRT techniques (ssIMRT, dMLC, VMAT) for head-and-neck and meningioma cases by means of PTV coverage, dose to OARs and some plan related parameters (MU, segments, treatment time).

One single person generated all plans using the same TPS version and dose algorithms, in order to keep the bias of this study low. Inevitably, differences are introduced by different optimization and sequencing algorithms needed for different techniques. Thus, these results are to some extent specific for the TPS (and its version) used in this study. By design of this study, the aim was to create plans where the dose to OARs within each case is comparable, thereby revealing the specific impact of MLC design and IMRT technique in terms of the target coverage. Thus, constrained optimization was used to find a Pareto optimal plan, which had all dose-limiting constraints minimized individually on a case-specific basis, such that the PTV coverage was just not affected for the VMAT^Agility^ plan. As ssIMRT and dMLC use static gantry angles, the chosen angles may affect the results. However, as the results show, dMLC^Agility^ plans degrade only slightly compared to VMAT^MLCi2−/+^, showing that the selected gantry angles were chosen reasonably with respect to PTV and OAR and therefore differences result from the used IMRT technique and MLC design.

As intended by constrained optimization, OAR exposure is within the prescriptions and therefore mainly the same for all 9 plans of each case (Figs. 2d and 3c). Dose differences within the prescribed limits are found, because the used prescription functions (modelling the biological effect of radiation to different tissues) do not match the clinical evaluated parameters. As the prescribing functions consider the organ exposure as a whole in terms of EUD (applying model specific parameters) and not in terms of single dose-volume-parameters, DVH curves may differ but result in the same EUD. Especially if critical structures are close to the PTV or belong partially to the PTV (larynx, parotid glands, mandibula, eyes), OAR exposure of a certain part of the DVH rises slightly with increased PTV coverage for the VMAT plans. However, as other parts of the same OAR can be preserved better by more complex techniques, the EUD remains the same. The higher OAR exposure for certain DVH parts found for more complex techniques is not only caused by higher scatter due to employing more beam directions and MU [31–33], but also due to the recently introduced advanced segment shape optimization that places leaves in VMAT optimization more effectively, such that constraints are not violated but PTV coverage is improved. By this, the distance between the projected leaf tip position and the OAR can be smaller for VMAT than for ssIMRT and dMLC.

The main results of this study show that (1) the smaller leaves of the Agility-MLC are capable of sparing the OARs to the same extent as with the MLCi2^+/−^, while increasing the PTV coverage, (2) using Elekta-VMAT planned with the TPS Hyperion V2.4 (equivalent to using Monaco 3.2) does not suffer the loss of either OAR sparing or PTV coverage and (3) Elekta-VMAT using the Agility-MLC planned with the TPS Hyperion V2.4 offers faster treatments than using the MLCi2^+/−^ or ssIMRT and dMLC.

Some authors [1, 5, 6, 15, 16, 33–37] studied the impact of different MLC properties showing 4 or 5 mm leaf width to be slightly superior over 10 mm leaf width in terms of either better PTV coverage, homogeneity and conformity or OAR sparing. Especially small OAR or target structures with small concavities will profit. As the aim of the presented study was to isolate differences in PTV coverage, homogeneity and conformity, narrower leaves give plans with higher mean dose to the target as well as improved homogeneity and conformity, for most cases. Consistent to van Kesteren *et al.* [15] and Lafond *et al.* [16], interdigitation had only little impact on improved PTV coverage, but could improve delivery efficiency by means of reduced MU [16], because in critical situations, OAR sparing can be performed more precisely and efficiently, if leaves can move freely. If interdigitation of the leaves is possible, the algorithm can choose the most efficient mode of sparing.

Higher impact on improved PTV coverage is found by technique. Shown by others [31, 33, 35, 38–42], comparable or slightly improved PTV coverage with better conformity and homogeneity is found comparing static or dynamic IMRT techniques to rotational IMRT. The recently introduced advanced segment shape optimization routine further improves homogeneity, especially for meningioma cases. Here, leaves are more effectively placed for better OAR sparing and better PTV coverage. Consequently, best PTV coverage is obtained for VMAT^Agility^ optimized with the advanced segment shape optimization routine.^1^

Forty to 50 % treatment time reduction is reported using VMAT instead of ssIMRT and MLCi2 for cases with medium or high PTV complexity (prostate, HN, lung) [31, 33, 38, 39, 42]. This current study also shows 37 to 50 % faster treatments for highly complex PTVs (HN) and 28 to 45 % for less complex PTVs (MG) using VMAT instead of ssIMRT. Wizorek *et al.* [43] report over 75 % faster treatments with RapidArc compared to dynamic IMRT for HN cases, whereas this study finds 13 and 30 % (MLCi2^−/+^ and Agility) for HN and 30 and 48 % (MLCi2^−/+^ and Agility) for MG. Reasons for this discrepancy of time speed up between the according Varian techniques (dynamic IMRT compared to RapidArc) on the one hand, and the Elekta techniques (dMLC compared to VMAT) on the other hand, may be manifold. First, RapidArc uses a different approach, in which the gantry speed modulation is considered to a smaller degree as for VMAT, mainly trying to keep gantry rotation speed at maximum. Second, this study uses two to three arcs and nine gantry angles instead of always two arcs and seven fields as Wizorek *et al.* Third, constraints are tighter in this study, resulting in more complex and therefore longer treatments. Forth, treatment times for dynamic IMRT in head-and-neck IMRT as reported by Wizorek *et al.* are at a higher level (10.5 ± 1 min) than treatment times calculated for this study using dMLC (7.0 ± 0.4 min). Therefore, a relatively higher gain in treatment time is possible. These higher treatment times for dMLC-IMRT calculated with Eclipse TPS is also shown by Shang *et al.* [44] (rectal cancer: 8.0 ± 0.7 min) and Jeong *et al.* [45] (HN: 11.7 to 19.6 min, depending on the number of fields).

The Agility-MLC accelerates treatments compared to MLCi2^−/+^ in this study by 22–26 % (HN) and 17–19 % (MG) for ssIMRT und dMLC, presumably due to higher leaf speed. Only evaluating VMAT, treatments are about 39 % faster for HN and meningioma. Bedford *et al.* [27] report 53 % accelerated VMAT treatments for typical HN. Tighter constraints in the presented study can cause longer treatment times for all techniques, and Bedford *et al.* use only one arc, a maximum delivery time constraint as well as a different optimization approach.

Even though reported treatment times are not measured but TPS-calculated and therefore only estimates, calculations were done with realistic assumptions for the dose rate, leaf and gantry rotation velocity. All these parameters were the same throughout the study. The absolute treatment times depend on the maximal achievable dose rate, leaf and gantry rotation velocity of a specific linac and may vary due to daily output variations, the actual wear of the single moving components as well as MLC calibration and beam tune. Hence, treatment times are comparable within this study and at least comparable in a relative manner to other studies.

A decrease in MU when treating HN with VMAT (1–2 arcs) instead of ssIMRT of 9 to 20 % has been reported [31, 33, 42]. Contrary, we find increased MU (10 % MLCi2^−^ (p = 0.06), 25 % MLCi2^+^ (p ≤ 0.01), 26 % Agility (p ≤ 0.01)) as also found by Guckenberger *et al.* [31] (7 % MLCi2^−^) when using three arcs for VMAT. Large MU reductions (35–60 %) compared to dMLC are reported for RapidArc [40, 43–47]. In the presented study, again, increased MU (10–19 %) are found if using VMAT instead of dMLC for HN cases. For MG, no significant MU reduction between dMLC and VMAT is found. One reason is that dMLC, calculated with Eclipse for Varian linacs, typically produces over 1100MU as compared to 800MU in this study, while for RapidArc less MU than for Elekta-VMAT are needed [40].

Burmeister *et al.* [1] found increased MU for narrower leaves while Wang *et al.* [36] report less MU for narrower leaves (4 mm). Also the presented data show improved MU efficiency with the narrower leaf MLC (Agility) for ssIMRT and VMAT. One reason could be that Burmeister normalized the plans. As in that study, homogeneity and D_min_ of the PTV was less for the narrower leaf plans, the normalization can involuntarily cause higher mean dose in the PTV and thereby higher MU. Besides, lower transmission leads to lower doses (esp. low dose region) in OARs [5] possibly enabling the constrained optimization algorithm to achieve higher PTV coverage for a given amount of extra dose due to MLC transmission. Therefore, presumably the lower transmission of the Agility-MLC compared to the MLCi2 is also one reason for improved MU efficiency.

## Conclusion

Best plans in terms of PTV coverage (EUD, D_mean_, HI, CI) while maintaining the OAR exposure and treatment delivery time are found for VMAT plans, delivered with the Agility-MLC. Due to reduction in transmission and improvements in leaf speed, these plans have only 3 and 16 % (HnN/meningioma) more MU than the corresponding ssIMRT, delivered with a non-interdigitating MLCi2.

## Abbreviations

CI: Conformity index
D_max_: Maximal dose (as dose to 99 % of the volume)
D_mean_: Mean dose
D_min_: Minimal dose (as dose to 1 % of the volume)
dMLC: Dynamic sliding window IMRT
DVH: Dose volume histogram
EUD: Equivalent uniform dose
Fig: Figure
GTV: Gross tumor volume
Gy: Gray
HI: Homogeneity index
HN: Head and neck
ICRU: International Commission on Radiation Units and Measurements
IMRT: Intensity modulated radiotherapy
MG: Meningioma
MLC: Multileaf collimator
MR: Magnet resonance
MU: Monitor units
OAR: Organ at risk
PET: Positron emmision tomography
PTV: Planning target volume
QUANTEC: Quantitative analysis of normal tissue effects in the clinic
SIB: Simultan integrated boost
ssIMRT: Step-and-shoot IMRT
Tab: Table
TPS: Treatment planning system
VMAT: Volumetric modulated arc therapy
V_xGy_: Volume receiving a dose of xGy

## Footnotes

1 This advanced segment shape optimization routine is also available for ssIMRT in Monaco 5.1 and higher, and for dMLC in Monaco 5.0 and higher. With this, improved plans for ssIMRT and dMLC will be found.
